# Fast and Flexible: Argentine Ants Recruit from Nearby Trails

**DOI:** 10.1371/journal.pone.0070888

**Published:** 2013-08-14

**Authors:** Tatiana P. Flanagan, Noa M. Pinter-Wollman, Melanie E. Moses, Deborah M. Gordon

**Affiliations:** 1 Department of Biology, University of New Mexico, Albuquerque, New Mexico, United States of America; 2 Department of Computer Science, University of New Mexico, Albuquerque, New Mexico, United States of America; 3 Department of Biology, Stanford University, Stanford, California, United States of America; University of Arizona, United States of America

## Abstract

Argentine ants (*Linepithema humile*) live in groups of nests connected by trails to each other and to stable food sources. In a field study, we investigated whether some ants recruit directly from established, persistent trails to food sources, thus accelerating food collection. Our results indicate that Argentine ants recruit nestmates to food directly from persistent trails, and that the exponential increase in the arrival rate of ants at baits is faster than would be possible if recruited ants traveled from distant nests. Once ants find a new food source, they walk back and forth between the bait and sometimes share food by trophallaxis with nestmates on the trail. Recruiting ants from nearby persistent trails creates a dynamic circuit, like those found in other distributed systems, which facilitates a quick response to changes in available resources.

## Introduction

Ant colonies operate without central control. The foraging behavior of an ant colony is a collective process [1] with dynamics that vary among species [2]. The dynamics that lead to the formation and maintenance of trails determine how well the colony selects and exploits available food sources [3].

Recruitment to food by ants has been studied for many decades (see e.g. [4]). Early work on recruitment showed that ants accumulate over time at food baits in response to direct and indirect social cues [5], [6] such as pheromone trails [7], tandem running [8], [9], and interactions among foragers, initiated by the ants that first encountered the food source. Diversity in recruitment strategies is probably related to the diverse ecological conditions in which colonies search for and retrieve food.

The Argentine ant *Linepithema humile* is an invasive species that has spread throughout the world [10]–[12], including northern California [13]–[15]. Colonies are polydomous, occupying at least two spatially distinct nests [16]. The network of separate nests, connected by persistent trails [17]–[19], spans up to 200 m^2^ in the summer and contracts to a few large aggregations in the winter [20]. As in many polydomous ant species [21], food [18], [22] and brood [10] are transported from one nest to another along the trails [23]. Argentine ants explore using a search process that links individual path shape to density [24], and lay pheromone trail as they move [25]. Rapid recruitment to food sources appears to provide Argentine ants with an ecological competitive advantage in its exotic range, because native species tend to retreat from food sources occupied by Argentine ants [26].

Many ant species exhibit central place foraging [1], [27], which may incur substantial travel costs when the foraging area is large, because each ant must travel back to a central nest [28]. Urban road networks, like ant trails, form branching structures to move individuals and resources. Cities often reduce per capita travel distances by using distributed transportation networks between dispersed locations without reliance on a single central transportation hub, reducing the costs of resource transport [29]. Similarly, in Argentine ants, recruitment from the pool of workers on nearby persistent trails could reduce travel costs, and increase the speed with which ants accumulate at a new food source. Here we investigate whether Argentine ants recruit nestmates directly from nearby trails.

**Figure 1:**
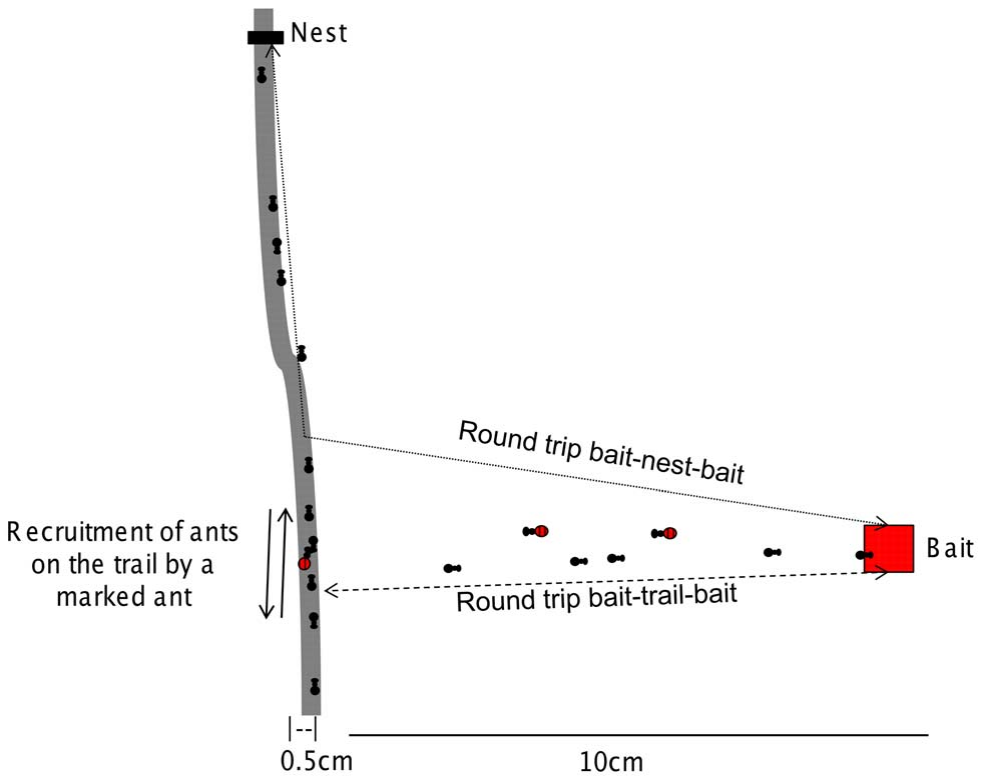
Experimental setup. The sketch represents our experimental setup. The thick gray line represents the persistent trail. The dotted line represents round trips taken by ants from the bait to the nest. The dashed line represents round trips taken from the bait to the trail. Marked ants that drank sugar water form the bait are shown with red, striped abdomens. Note the marked ant on the trail that goes back and forth recruiting nestmates.

Our field trials test whether Argentine ants recruit workers to new food sources from persistent trails. We observe the recruitment behavior of ants at bait and examine whether recruited ants come from the pool of workers already available on a nearby persistent trail or from a nearby nest (Fig. 1).

## Materials and Methods

We studied the foraging behavior of Argentine ants on the Stanford University campus near Palo Alto, California, from May 16–26, 2011. In spring and summer, the mean distance between Argentine ant nests linked by trails is about 15 m [20]. We performed 13 baiting trials on two persistent trails, on opposite sides of a building, with 5 trials for the trail on the West and 8 trials for the trail on the East side of the building. The trails were confined to tight spaces between concrete blocks and were never wider than 0.5 cm. Here we examine a) whether Argentine ants recruit from the trail and b) demonstrate (quantitatively) how fast this recruitment occurs.

### Experimental setup

Trails were visible in the cracks between large paving stones. For each trial, baits were placed for 90 minutes approximately 10 cm from a persistent trail (Fig. 1). The distance between trail and baits was an experimental constraint imposed by the ants' behavior. We observed that the ants do not reliably find a bait that is more than 0.3 m away from the trail in less than a day. Thus, we chose this distance because ants appeared at the baits at this distance within 12 hours. We recorded all activity during this period. The bait consisted of sugar water in a concentration of 25% sugar to water volume. We saturated a 2×2 cm^2^ square piece of cotton in sugar water and placed it on top of a paper towel of approximately the same size. To mark the ants that visited the bait, we added four drops of food coloring (Americolor Soft Gel Paste) to the solution [as in 20].

We filmed the bait for the 90-min duration of each trial using a JVC GZ-HM670 HD Everio Camcorder. An ant was considered to have arrived at the bait when it started drinking from the cotton or paper towel. When an Argentine ant ingests colored water its abdomen swells, making the colored water visible between abdominal segments. We distinguished between arrivals of unmarked ants that had not yet ingested the bait, and arrivals of ants with a colored abdomen that had previously ingested the bait during that trial.

Data were recorded using an iPad app that we wrote called EventLog.

### Origin of new arrivals

To investigate whether recruitment occurred from the pool of available workers on the persistent trail or from workers at the nest we followed individual ants as they left the bait. We haphazardly selected 2–7 ants in each trial that were ingesting the bait, and observed the ants as they returned to the trail and then went back to the bait. Only few marked ants were present simultaneously in the area between the bait and the trail, thus there was little chance of confusing the identity of marked ants. We recorded the time it took each ant to reach the trail, the time it spent at the trail, and the time it took to return to the bait.

We defined a ‘bait-trail-bait’ round trip as the time for an ant to go from the bait to the trail and back, and compared this time to the ‘bait-nest-bait’ round trip, the time for an ant to go from the bait to the nearest possible nest location and back to the bait (Fig. 1). We obtained a direct measure of individual ‘bait-trail-bait’ round trip times by following individual ants as they walked from the bait to the trail and back. We call this measure ‘observed’ bait-trail-bait round trip time.

To calculate the ‘bait-nest-bait’ round trip time, we first measured the speed of ants walking on the trail by selecting 2–6 ants during each trial and measuring the time it took each ant to walk one meter. We estimated the mean time to the nest as distance divided by velocity. To locate the nearest possible nest location we followed trails until we found ants disappearing under paving stones. The concrete was light colored, contrasting well with the dark color of the ants, making it easy to see the ants. To calculate a minimum time to return to and from the nest, we considered the nearest point where the persistent trail disappeared and the ants could have been entering a nest. Our ‘bait-nest-bait’ round trip times may be underestimated because a nest could have been located further from the location where the ants disappeared under the paving stones.

To test whether ants returned to the bait without first returning to the nest, we compared bait-trail-bait round trip times with bait-nest-bait round trip times using ANOVA. We additionally measured the round trip time for the first marked ant that returned in each trial and defined it as ‘estimated’ bait-trail-bait round trip time. We compared that time to the ‘observed’ bait-trail-bait time and to the bait-nest-bait time.

### Recruitment

We tested for recruitment in two ways. First, following [30] and [31], we measured the change in the number of ants at the bait over time. To test whether arrival at the bait was due to recruitment rather than to chance, we examined the relationship of the flow of ants on the trail with the number of ants on the bait. Second, to test whether the rate at which new ants arrived at the bait increased, we used a regression. We tested whether the increase in arrival rates was due to recruitment by comparing it with the rate discovery by chance.

To determine if the number of ants at the bait increased over time, we recorded the time of arrival of each ant at the bait, and subsequently counted the number of ants on the cotton and the paper towel throughout the trial, approximately every 5 minutes.

We measured the flow of ants on the trail by counting the number of ants passing an invisible line on the trail, in both directions, for one minute, 3–9 times for each trial. If ants arrive at a bait alongside a persistent trail by chance alone, then the rate of arrival of ants at the bait should be correlated with the rate of flow of ants on the persistent trail; more ants moving along the persistent trail would lead more ants to discover the bait. However, if ants actively recruit nestmates from the trail, then the rate of arrival at the bait would not necessarily be correlated with the rate at which ants are moving along the persistent trail.

We examined the relationship between the number of ants at the bait and the flow of ants on the persistent trail in several ways. First we used a linear regression to directly compare the flow of ants on the persistent trail as the dependent variable to the number of ants at the bait as the independent variable. To allow for a comparison between the number of ants on the bait and the flow of ants on the persistent trail, we used a one-dimensional data interpolation to produce continuous data points for the flow variable. We then examined how the relationship between the number of ants on the bait and the flow of ants on the persistent trail depended on time. We tested for positive slopes in the linear regression of each of these two variables versus time. We then normalized the number of ants on the bait by dividing it by the flow on the persistent trail and calculated a linear regression of this ratio against time. We used the False Discovery Rate (FDR) [32] to correct for multiple testing.

We defined rate of arrival as the time between new arrivals to the bait and the time to discover the bait by chance as the time it took the first ant to discover the bait in each trial. We examined increasing arrival rates by using an exponential regression with the time between arrivals at the bait of new, unmarked ants, as the dependent variable and the cumulative number of new ants that had arrived at the bait as the independent variable. We used an exponential regression instead of a linear regression because it provided a better fit to the data when comparing the two models using the Akaike Information Criterion (AIC) [33].

To test whether the rate of arrival at the bait was faster than random discovery, we compared the time between arrivals at the bait with the time it took for the first ant to discover the bait. We expected recruitment to cause ants to arrive at the bait faster than the time it took the first ant to discover the bait. We define ‘discovery time’ as the time it took the first unmarked ant to discover the bait and used it to estimate how long it would take an ant to arrive at the bait by chance. Recruitment should decrease the time between successive arrivals of new ants at the bait.

All means are reported ± standard deviations. Analysis was conducted using Matlab (7.12.0.635 R2011a, Mathworks, MA) and IBM SPSS Statistics (Version 20, NY).

## Results

Ants are recruited from the persistent trail. Of the 47 ants followed as they left the bait and returned to the trail, we observed that 40% (19 ants: 5 of 15 followed on the East trail and 14 of 32 followed in the West trail) completed a second trip, returning from the trail to the bait and back to the trail again. Once on the trail, the recruiting ants spent 0.87±0.67 minutes interacting with nestmates. We observed these ants sharing sugar water with a few of their nestmates on the persistent trail, via trophallaxis, before returning to the bait. The mean round trip time from the bait to the trail and back again for the 19 ants that were followed for the entire round trip was 1.78±1.46 minutes (East trail: 1.38±0.67, West trail: 1.92±1.66 minutes). We lost visual contact with the remaining ants (60%) once they arrived at the trail.

**Figure 2:**
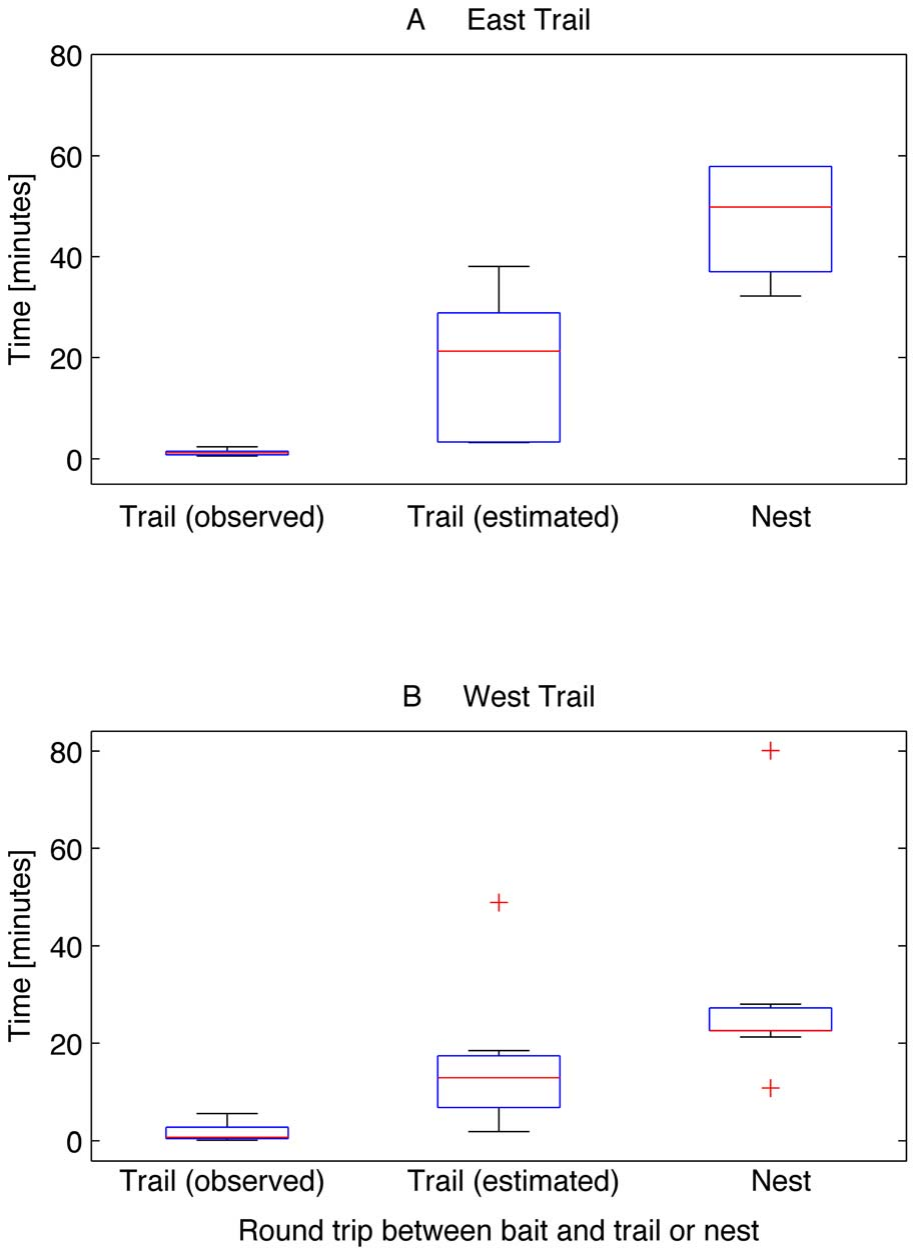
Round trip ‘bait-trail-bait’ and ‘bait-nest-bait’ durations. The time to complete a round trip from the bait to the nest and back is significantly longer (*p*<0.001) than the observed and estimated bait-trail-bait time for both East and West trails. Box plots represent the duration for (A) seven measurements on the East trail, and (B) seventeen measurements on the West trail. The central line on each box is the median. The box edges are the 25th and 75th percentiles. The whiskers extend to the most extreme data points that are not considered outliers which are plotted individually as “+”.

Recruitment from the persistent trail is faster than recruitment from the nest. The minimum distance to the nest for the East trail was 17.88 meters; and for the West trail, 12.19 meters. We estimated the mean speed of ants on the trail as 1.07±0.45 meters per minute for the East trail and 1.06±0.49 meters per minute for the West trail. Thus, ants from the East trail required at least 30.52±18.91 minutes to travel from the bait to the nest and back, and ants from the West trail could complete this round trip in 18.91±13.77 minutes. In all trials, observed and estimated bait-trail-bait round trip times were significantly shorter than the mean bait–nest-bait trip times (East trail ANOVA F = 20.82 N = 7, *p*<0.001; West trail ANOVA F = 18.48 N = 17 p<0.001) (Fig. 2).

**Figure 3:**
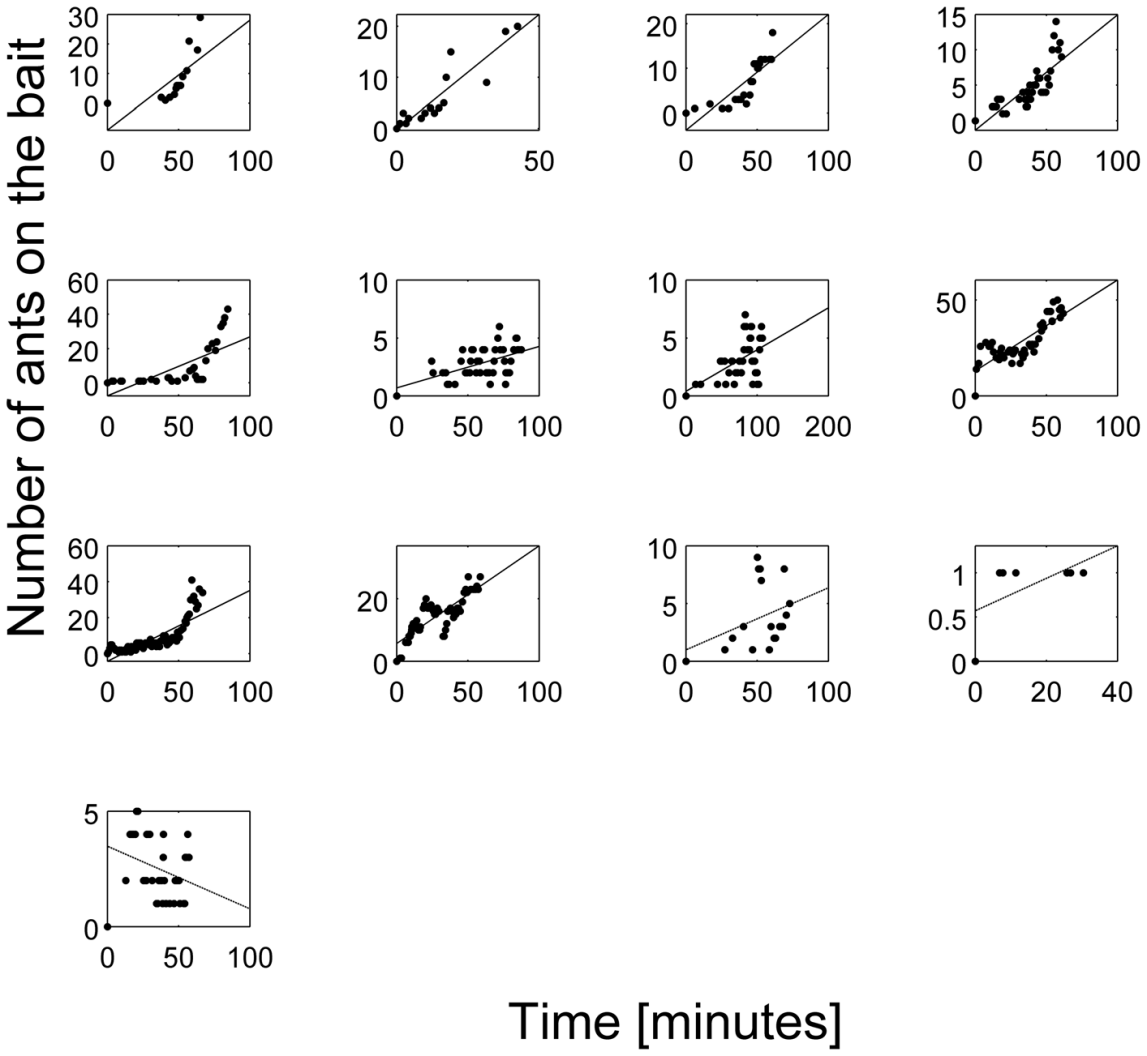
Number of ants on the bait over time. Each plot represents one trial. Ten of thirteen trials have a significant positive slope (first two rows, Table 1). The regressions for the last three trials are not significant. Solid lines represent significant linear regressions (*p*<0.01). Dashed lines represent linear regressions that are not significant (*p*>0.05).

The number of ants at the bait increased significantly over the course of the trial. Ten of the thirteen trials showed a positive and significant (*p*<0.01) slope and three were not significant (*p*>0.05) (Fig. 3, Table 1). Both trails exhibited an increase in the number of ants at the bait over time. The slopes of linear regressions on values from all trials were both significantly positive (0.13 East and 0.08 West, *p*<0.05).

**Table 1:** Statistics for linear regressions.

		# Ants on bait vs. time			Flow on trail vs. time			# Ants on bait/flow on trail vs. time			# Ants on bait vs. flow on trail			Inter arrival time vs. # ants on bait		
Trail	Trial	R^2^	P	Slope	R^2^	P	Slope	R^2^	P	Slope	R^2^	P	Slope	R^2^	P	Slope
East	1	**0.46**	**0.007**	**0.13**	0.22	0.200	0.13	**0.84**	**<0.001**	**0.02**	**0.57**	**0.003**	**−1.11**	**0.13**	**<0.001**	**<0.01**
East	2	**0.82**	**<0.001**	**0.03**	0.01	0.888	0.03	**0.78**	**<0.001**	**0.01**	0.02	0.640	0.17	0.04	0.058	**−**0.01
East	3	**0.70**	**<0.001**	**0.28**	0.10	0.601	0.28	**0.67**	**<0.001**	**<0.01**	**0.50**	**<0.001**	**0.25**	**0.36**	**0.019**	**−0.51**
East	4	**0.62**	**<0.001**	**0.18**	<0.01	0.460	0.18	<0.01	0.847	<0.01	0.01	0.587	0.01	**0.06**	0.497	**−**0.40
West	5	**0.50**	**<0.001**	**0.01**	<0.01	0.961	0.01	**0.36**	**<0.001**	**0.01**	0.03	0.369	**−**0.29	**0.18**	**<0.001**	**−0.02**
West	6	**0.28**	**<0.001**	**0.12**	0.11	0.378	0.12	**0.18**	**0.004**	**<0.01**	0.02	0.405	0.03	**0.17**	**0.026**	**−0.11**
West	7	**0.26**	**0.001**	**0.23**	0.58	0.047	0.23	**0.15**	**0.014**	**<0.01**	<0.01	0.842	0.01	**0.26**	**<0.001**	**−0.08**
West	8	**0.66**	**<0.001**	**0.37**	0.35	0.042	0.37	**0.08**	**0.027**	**<0.01**	**0.24**	**<0.001**	**0.42**	**0.02**	**0.001**	**<0.01**
West	9	**0.64**	**<0.001**	**0.08**	0.01	0.845	0.08	**0.76**	**<0.001**	**<0.01**	**0.33**	**<0.001**	**0.23**	<0.01	0.812	**−**0.01
West	10	**0.70**	**<0.001**	**0.08**	<0.01	0.905	0.08	**0.48**	**<0.001**	**0.01**	<0.01	0.718	0.01	0.14	0.402	**−**0.40
West	11	0.12	0.147	0.14	0.13	0.428	0.14	0.06	0.331	<0.01	0.19	0.066	**−**0.13	**0.12**	**0.018**	**−0.04**
West	12	0.33	0.174	0.45	0.68	0.087	0.45	0.02	0.772	<0.01	0.22	0.345	0.02	**1.00**	**<0.001**	**−12.1**
West	13	0.08	0.068	**−**0.14	0.13	0.491	**−**0.13	**0.20**	**0.008**	**<0.01**	0.06	0.164	0.07	0.09	0.400	**−**0.57

Statistical indicators for goodness of fit and slopes for linear regressions for number of ants on the bait, flow of ants on the trail and time between arrivals. Bold numbers indicate statistically significant values after FDR-corrected for multiple comparisons.

The increase in the number of ants on the bait appears to be the result of active recruitment of nestmates from the persistent trail, not random arrivals due to an increased flow on the persistent trail. The flow of ants on the persistent trail did not change over time, none of the regressions showed a significant relationship between number of ants at the bait and flow on persistent trail (Table 1). When we divided the number of ants at the bait by the flow of ants on the persistent trail, 10 of 13 trials showed a significant (p<0.05) increase against time (Table 1).

**Figure 4:**
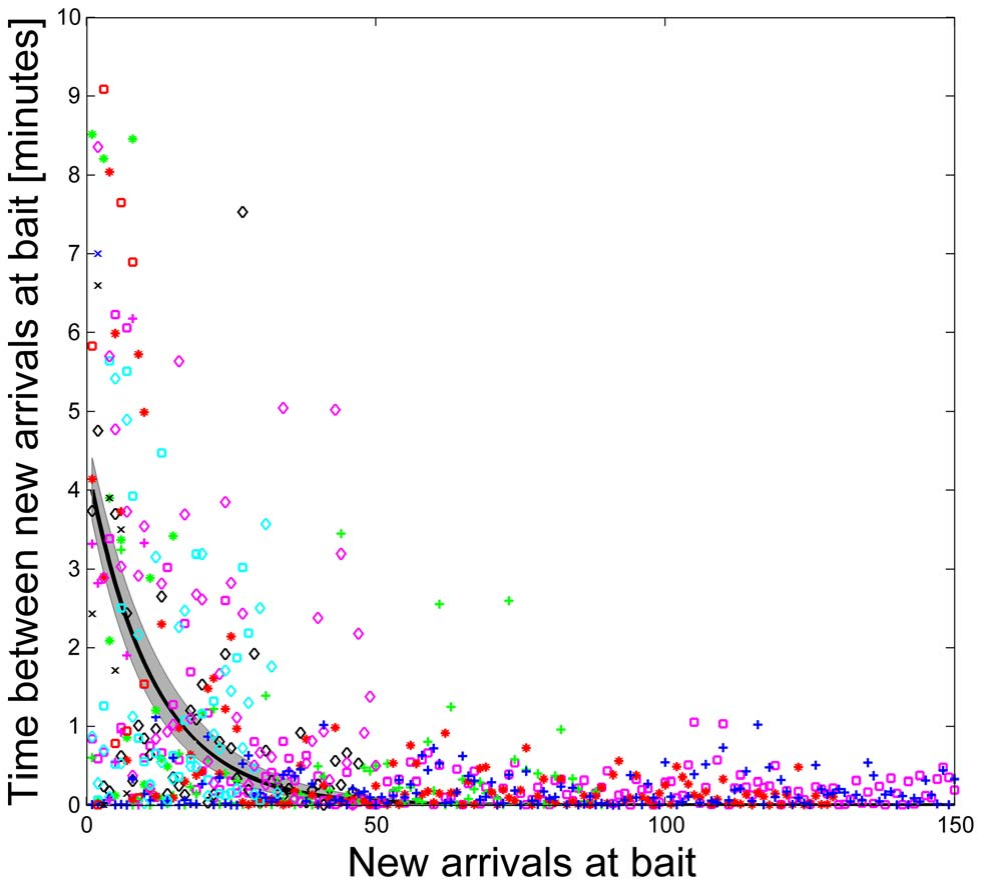
Time between ant arrivals at the bait versus cumulative number of new ants that have arrived at the bait. The exponential regression shows a rapid decrease in time between arrivals at the bait. As more ants arrive at the bait, the time between arrivals decreases. Data for each trial are represented by a symbol of a different color and shape. Exponential regression is shown as a thick black line. The shaded area represents the 95% confidence interval.

Arrival at the bait provides positive feedback that leads to more arrivals at the bait. As the number of new arrivals at the bait increases, the time between successive arrivals decreases exponentially (Fig. 4), SSE  = 2299, R^2^ = 0.35, RMSE  = 1.26). In 8 of 13 trials, the time between arrivals of new ants at the bait decreases significantly (*p*<0.05) as the number of ants that visit the bait increases (Table 1).

Arrival at the bait occurs more rapidly than it would if the bait were discovered by chance (Fig. 2). The time between new arrivals is substantially shorter than the time between bait placement and first bait discovery (two sided Wilcoxon Signed Ranks Test, Z = 6.09, N_arrivals_  = 1467, N_discoveries_  = 13, p<0.001).

## Discussion

We found that Argentine ants recruit nestmates to food bait from persistent trails. After locating a bait placed near a persistent trail, recruiting ants returned to the trail, and some shared food with nestmates on the trail. Activity at the bait, measured as the number of ants and the rate of arrival at the bait, increased as a result of recruitment, not as a result of an increase in the flow of ants on the persistent trail.

We directly observed 40% of marked ants going from the bait to the trail and back to the bait. This may be an underestimate because we do not know what fraction of the ants we did not directly follow went only to the trail or returned to the nest. Further, the time it takes an ant to complete a round trip from the bait to the nest and back is significantly longer than the durations of round trips that we estimated for the first ants to appear at the bait.

The increase in number of ants at the bait over time was not due to an increase in the flow of ants on the persistent trail. The time between arrivals at the bait decreased exponentially, indicating positive feedback due to recruitment (Fig. 4). We found that the intervals between the arrivals of subsequent foragers at the bait were shorter than the time it took the first forager to discover the bait initially, indicating that recruitment, rather than chance discovery, accounts for the increase in number of ants at the bait. Further work is needed to learn what determines the intervals between arrivals at the bait, and how these lead to non-linear accumulations of ants at baits [34].

Our findings are based on observations of ants that take up colored sugar water into their gaster. Previous work [22] showed that 80% of Argentine ants that ingested colored dye remain visibly marked after 14 days even though they engaged in trophallaxis. Therefore, it seems unlikely that many marked ants lost the color through transfer to nestmates in our 1.5-hour trials, so the unmarked ants were probably new arrivals at the bait.

A high proportion (40%) of the ants that we followed, after they found and fed from the bait, went back and forth from the trail to the bait. These ants were probably depositing a pheromone trail, but this remains to be demonstrated. Some species use different pheromones to signal different types of trails. Nelson et al. [35] discussed the possible use of different pheromones for main and side trails in *Paraponera clavata*. *Pheidole megacephala* use two different pheromones, a long-lasting pheromone to explore and a short-lasting pheromone to recruit to a food source [36]. Similarly, *Anoplolepis gracilipes* [37] and *Paratrechina longicornis* [38] use short- and long-lasting pheromone signals. Aron et al. [25] showed that Argentine ants lay a chemical trail as they walk. Additional work to explore the use of multiple recruitment pheromones in Argentine ants could determine whether these ants use different pheromones for persistent versus ephemeral trails.

Further work is needed to determine which ants are more likely to leave a persistent trail for a new food source, whether the small trails formed by short-term recruitment later become larger, more persistent trails [20], and how these dynamics are related to the quality and duration of the food source. Forming branches from persistent trails to exploit an ephemeral food source may expedite foraging and increase colony efficiency in obtaining food. This previously undescribed behavior, recruitment from a persistent trail, uncouples information about the location of a food source from the transport of the food to the nest. Further work is needed to determine how often, and under what conditions, Argentine ants employ recruitment from existing persistent trails. Additionally, because our study was limited to two trails that were 50 m from one another, close enough that they could conceivably have belonged to the same colony, future studies should examine differences in local recruitment behavior among multiple colonies and ecological conditions.

The flexible recruiting system we describe, allowing new trails to form from worker pools available in nearby persistent trails, may help account for this species' success as an invader [39], [40]. Recruitment from nearby trails accelerates recruitment and food collection by a factor of at least 9 (Fig. 2). Argentine ants are successful in competing with native species in many areas of their invasive range, in part because they arrive at food sources more quickly than native species [22], [26]. Similar dynamics can be found in other mass recruiters. For example, the trail formation by means of local recruitment can be compared to the exploratory dynamics of *Monomorium pharaonis*, whose workers interact through the trail pheromone laid on the ground and lead to the emergence of a network of exploratory trails from which scouts coming back from a food source can recruit [41]. *Lasius niger*, use short-term exploratory trails to guide workers towards rich food sources [42] and *Leptogenys ocellifera* use permanent and ephemeral routes that may change according to the food supply [43]. While flexible, ephemeral trail formation has been observed in other species, the novelty of our findings is that we demonstrate recruitment directly from persistent trails rather than recruitment from the nest as in, *Paraponera clavata* or from within a foraging area as in the Brazilian ant *Pheidole oxyops* [44].

The Argentine ant strategy of recruitment from the trail suggests a solution to a common engineering problem, that of collecting or distributing resources in “the last mile.” At the last mile, infrastructure networks connect to individual consumers. The last mile can be wired, such as cables that connect individual homes to trunk lines, or wireless where a tower connects cell phones to a high-speed backbone. In biological and engineered networks, the dynamics in the last mile can set the pace of the entire system [45]. The last mile presents a challenge, because if a network delivers or collects resources in a large area, the majority of the network wires may be in the many short-distance low-capacity links that fill the last mile.

Just as virtual networks like cell phone towers make coverage of the last mile less difficult than constructing permanent wired networks, the ephemeral recruitment trails that appear in response to newly discovered food and connect to more established, persistent trails provide an efficient way of routing resources in Argentine ants. Ants that discover new food, and go to a persistent trail to communicate that discovery to other ants, act as relays that efficiently route ants to ephemeral food. Although trails between nests are always needed for movement between nests, the ephemeral relays to local baits provide a fast and flexible mechanism for routing ants from persistent trails to ephemeral food. The network exists only when it is needed, and when the resource is exhausted, the network can disappear so that effort can be invested elsewhere. Ants have evolved solutions to many searching and communication problems [46], [47] that mirror or inspire approaches used by engineers. The ability of Argentine ants to cover the last mile with ephemeral trails is yet another example.

## References

[1] Traniello JFA (1989) Foraging strategies of ants. Annual Review of Entomology 34: 191–210.

[2] Beckers R, Goss S, Deneubourg JL, Pasteels J (1989) Colony size, communication, and ant foraging strategy. Psyche 96: 239–256.

[3] Camazine S, Deneubourg J, Franks N, Sneyd J, Theraulaz G, et al.. (2001) Self-Organization in Complex Systems. Princeton University Press, Princeton.

[4] Wilson EO (1962) Chemical communication among workers of the fire ant *Solenopsis saevissima* (Fr. Smith) 2. An information analysis of the odour trail. Animal Behaviour 10: 148–158.

[5] Detrain C, Deneubourg J (2009) Social cues and adaptive foraging strategies in ants. Food Exploitation by Social Insects: Ecological, Behavioral, and Theoretical Approaches Boca Raton, USA: CRC Press: Contemporary Topics in Entomology Series: 29–52.

[6] Gordon DM (2010) Ant Encounters: Interaction Networks and Colony Behavior: Princeton University Press.

[7] Hölldobler B, Lumsden CJ (1980) Territorial strategies in ants. Science 210: 732. 17739532 10.1126/science.210.4471.732

[8] Franks NR, Richardson T (2006) Teaching in tandem-running ants. Nature 439: 153–153. 16407943 10.1038/439153a

[9] Fernandez AA, Deneubourg JL (2011) On following behaviour as a mechanism for collective movement. Journal of Theoretical Biology 284: 7–15. 21679718 10.1016/j.jtbi.2011.06.001

[10] Suarez AV, Holway DA, Case TJ (2001) Patterns of spread in biological invasions dominated by long-distance jump dispersal: insights from Argentine ants. Proceedings of the National Academy of Sciences 98: 1095. 10.1073/pnas.98.3.1095 PMC14714 11158600

[11] Carpintero S, Reyes-López J, Arias de Reyna L (2005) Impact of Argentine ants (*Linepithema humile*) on an arboreal ant community in Donana National Park, Spain. Biodiversity and Conservation 14: 151–163.

[12] Rowles AD, Silverman J (2009) Carbohydrate supply limits invasion of natural communities by Argentine ants. Oecologia 161: 161–171. 19452171 10.1007/s00442-009-1368-z

[13] Holway DA, Suarez AV (1999) Animal behavior: an essential component of invasion biology. Trends in Ecology & Evolution 14: 328–330. 10407433 10.1016/s0169-5347(99)01636-5

[14] Sanders NJ, Barton KE, Gordon DM (2001) Long-term dynamics of the distribution of the invasive Argentine ant, *Linepithema humile*, and native ant taxa in northern California. Oecologia 127: 123–130. 28547163 10.1007/s004420000572

[15] Knight RL, Rust MK (1990) Repellency and efficacy of insecticides against foraging workers in laboratory colonies of Argentine ants (Hymenoptera: Formicidae). Journal of Economic Entomology 83: 1402–1408.

[16] Debout G, Schatz B, Elias M, McKey D (2007) Polydomy in ants: what we know, what we think we know, and what remains to be done. Biological Journal of the Linnean Society 90: 319–348.

[17] Newell W, Barber T (1913) The Argentine ant. US Department of Agriculture Bureau of Entomology Bulletin.

[18] Markin GP (1968) Nest relationship of the Argentine ant, *Iridomyrmex humilis* (Hymenoptera: Formicidae). Journal of the Kansas Entomological Society: 511–516.

[19] Markin GP (1970) The seasonal life cycle of the Argentine ant, *Iridomyrmex humilis* (Hymenoptera: Formicidae), in southern California. Annals of the Entomological Society of America 63: 1238–1242.

[20] Heller NE, Gordon DM (2006) Seasonal spatial dynamics and causes of nest movement in colonies of the invasive Argentine ant (*Linepithema humile*). Ecological Entomology 31: 499–510.

[21] Cherix D, Bourne J (1980) A field study on a super-colony of the red wood ant *Formica lugubris* Zett. in relation to other predatory arthropodes (spiders, harvestmen and ants). Revue Suisse de Zoologie 87: 955–973.

[22] Heller N, Ingram K, Gordon D (2008) Nest connectivity and colony structure in unicolonial Argentine ants. Insectes Sociaux 55: 397–403.

[23] Latty T, Ramsch K, Ito K, Nakagaki T, Sumpter DJT, et al (2011) Structure and formation of ant transportation networks. Journal of The Royal Society Interface 8: 1298–1306. 10.1098/rsif.2010.0612 PMC3140716 21288958

[24] Gordon DM (1995) The development of an ant colony's foraging range. Animal Behaviour 49: 649–659.

[25] Aron S, Pasteels J, Deneubourg J (1989) Trail-laying behaviour during exploratory recruitment in the argentine ant, *Iridomyrmex humilis* (Mayr). Biology of Behaviour 14: 207–217.

[26] Human K, Gordon D (1999) Behavioral interactions of the invasive Argentine ant with native ant species. Insectes Sociaux 46: 159–163.

[27] Hölldobler B, Wilson EO (1990) The Ants: Belknap Press.

[28] Moses ME (2005) Metabolic scaling, from insects to societies. Ph.D. dissertation, University of New Mexico.

[29] Samaniego H, Moses ME (2008) Cities as organisms: Allometric scaling of urban road networks. Journal of Transport and Land use 1.

[30] Pratt SC, Mallon EB, Sumpter DJ, Franks NR (2002) Quorum sensing, recruitment, and collective decision-making during colony emigration by the ant *Leptothorax albipennis* . Behavioral Ecology and Sociobiology 52: 117–127.

[31] Hölldobler B (1976) Recruitment behavior, home range orientation and territoriality in harvester ants, *Pogonomyrmex* . Behavioral Ecology and Sociobiology 1: 3–44.

[32] Benjamini Y, Drai D, Elmer G, Kafkafi N, Golani I (2001) Controlling the false discovery rate in behavior genetics research. Behavioural Brain Research 125: 279–284. 11682119 10.1016/s0166-4328(01)00297-2

[33] Akaike H (1974) A new look at the statistical model identification. Automatic Control, IEEE Transactions on 19: 716–723.

[34] Detrain C, Deneubourg JL, Pasteels JM (1999) Information Processing in Social Insects: Birkhäuser.

[35] Nelson C, Jorgensen C, Black H, Whiting J (1991) Maintenance of foraging trails by the giant tropical ant *Paraponera clavata* (Insecta: Formicidae: Ponerinae). Insectes Sociaux 38: 221–228.

[36] Dussutour A, Nicolis SC, Shephard G, Beekman M, Sumpter DJ (2009) The role of multiple pheromones in food recruitment by ants. The Journal of Experimental Biology 212: 2337–2348. 19617426 10.1242/jeb.029827

[37] Lizon à l'Allemand S, Witte V (2010) A sophisticated, modular communication contributes to ecological dominance in the invasive ant *Anoplolepis gracilipes* . Biological Invasions 12: 3551–3561.

[38] Witte V, Attygalle AB, Meinwald J (2007) Complex chemical communication in the crazy ant *Paratrechina longicornis* Latreille (Hymenoptera: Formicidae). Chemoecology 17: 57–62.

[39] Holway DA (1999) Competitive mechanisms underlying the displacement of native ants by the invasive Argentine ant. Ecology 80: 238–251.

[40] Tremper BS (1976) Distribution of the Argentine ant, *Iridomyrmex humilis* Mayr, in relation to certain native ants of California: ecological, physiological, and behavioral aspects: University of California, Berkeley.

[41] Fourcassie V, Deneubourg J-L (1994) The dynamics of collective exploration and trail formation in *Monomorium pharaonis*: experiments and model. Physiological Entomology 19: 291–300.

[42] Beckers R, Deneubourg J-L, Goss S (1992) Trail laying behaviour during food recruitment in the ant *Lasius niger* (L.). Insectes Sociaux 39: 59–72.

[43] Maschwitz U, Mühlenberg M (1975) The strategy of predation in some oriental *Leptogenys* species. Oecologia 20: 65–83. 28309298 10.1007/BF00364322

[44] Czaczkes TJ, Ratnieks FLW (2012) Pheromone trails in the Brazilian ant *Pheidole oxyops*: extreme properties and dual recruitment action. Behavioral Ecology and Sociobiology: 1–8.

[45] Banavar JR, Moses ME, Brown JH, Damuth J, Rinaldo A, et al (2010) A general basis for quarter-power scaling in animals. Proceedings of the National Academy of Sciences 107: 15816–15820. 10.1073/pnas.1009974107 PMC2936637 20724663

[46] Prabhakar B, Dektar KN, Gordon DM (2012) The regulation of ant colony foraging activity without spatial information. Plos Computational Biology 8: e1002670. 22927811 10.1371/journal.pcbi.1002670 PMC3426560

[47] Dorigo M, Gambardella LM, Birattari M, Martinoli A, Poli R, et al.. (2006) Ant Colony Optimization and Swarm Intelligence: 5th International Workshop, ANTS 2006, Brussels, Belgium, September 4–7, 2006, Proceedings: Springer.

